# Panvascular concept in the evaluation and treatment of intracranial atherosclerotic stenosis

**DOI:** 10.3389/fneur.2024.1460124

**Published:** 2024-12-24

**Authors:** Jiahao Tang, Guoyang Zhou, Yuexin Lu, Shunan Shi, Lin Cheng, Jianping Xiang, Shu Wan, Ming Wang

**Affiliations:** ^1^The Second School of Clinical Medicine, Zhejiang Chinese Medical University, Hangzhou, China; ^2^Zhejiang University School of Medicine, Hangzhou, China; ^3^Brain Center, Zhejiang Hospital, Hangzhou, China; ^4^ArteryFlow Technology Co., Ltd., Hangzhou, China

**Keywords:** panvascular medicine, intracranial atherosclerotic stenosis, coronary artery disease, plaque, fractional flow reserve

## Abstract

Cerebrovascular disease is the leading causes of death and disability worldwide. Intracranial atherosclerotic stenosis (ICAS) is one of the major causes of ischemic stroke, especially in the Asian population. It is urgent to explore effective screening methods for early diagnosis to improve prognosis of patients with ICAS. Recently, the concept of panvascular medicine has provided a direction for the exploration of evaluation of ICAS. Based on the concept of “panvascular medicine,” atherosclerosis is the common pathological feature of panvascular disease, such as ICAS and coronary artery disease (CAD). In-depth research on the formation and development of plaques, the development and application of more precise preoperative assessment and detection methods, and the utilization of new interventional equipment have greatly enhanced the precision of diagnosis and treatment of CAD. Studies attempt to apply similar evaluation and treatment in ICAS. The deeper understanding, the more accurate diagnosis and treatment, contributing to improve the prognosis of patients with ICAS. This review focuses on these evaluations and treatment of CAD applied in the field of ICAS.

## 1 Introduction

**Figure 1:**
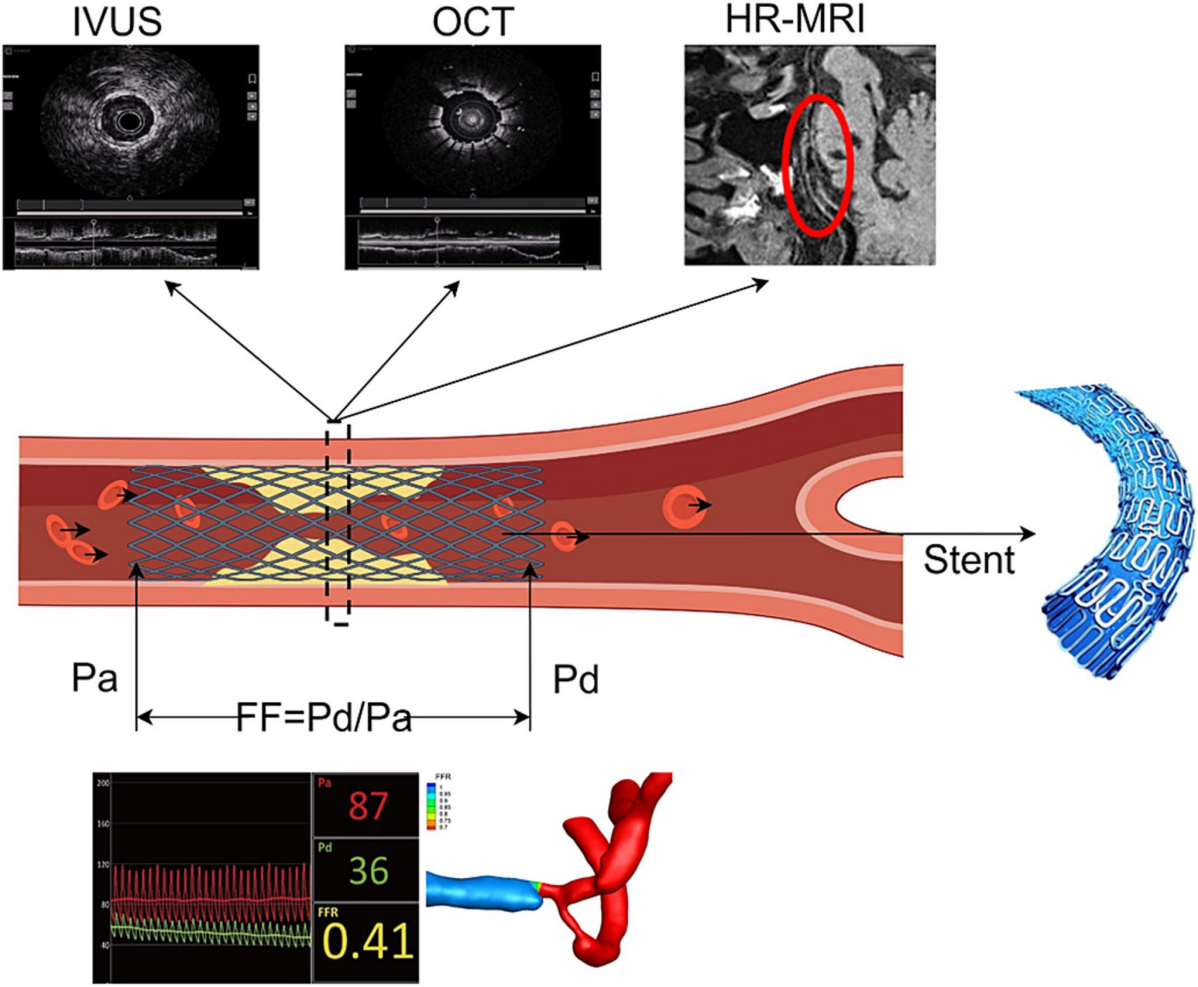
Schematic diagram of the related technologies in this review (by Figdraw).

Intracranial atherosclerotic stenosis (ICAS) is a major cause of ischemic stroke. Previous clinical trials have regarded medical therapy as the primary treatment approach for ICAS. However, even with aggressive medical management, the one-year stroke recurrence rate among ICAS patients remains at 12–15% (1, 2). Recently, the CASSISS clinical trial from China has demonstrated that the addition of percutaneous transluminal angioplasty and stenting to medical therapy is not inferior to medical therapy alone (3). This suggests that with more precise preoperative assessments, stringent patient selection criteria, and the utilization of advanced therapeutic devices, the clinical outcomes of interventional treatment may continue to improve. As the situation of vascular disease prevention and control becomes increasingly severe, the limitations of research focused on single diseases are gradually becoming apparent. Scholars have begun to pay attention to the systemic and common pathological features of such diseases, deepening their understanding of vascular diseases from the “vascular tree” to the “vascular network.” In 2002, Lanzer and Topol, based on a systematic and comprehensive understanding of vascular diseases, first proposed the concept of “pan-vascular disease” (4). These diseases share a common pathological characteristic of atherosclerosis, primarily affecting vital organs such as the heart, brain, kidneys, limbs, and major arteries. Due to the presence of similar or identical risk factors and pathological processes, the evaluation and treatment between different organs can be mutually informative. In the study of coronary atherosclerosis, technologies such as intravascular ultrasound (IVUS) and optical coherence tomography (OCT) have been widely used for plaque component identification, while fractional flow reserve (FFR) and wall shear stress (WSS) can accurately assess hemodynamics (5, 6). These technologies have achieved significant results in clinical applications. Building on this foundation, researchers have applied their experiences from coronary arteries to renal and peripheral arteries, confirming the value of hemodynamic parameters such as fractional flow reserve (FFR) in assessing stenosis in these areas (7–9). This provides guidance for addressing post-stent restenosis and further validates the feasibility of the “pan-vascular disease” concept. Based on this concept, scholars have begun to attempt to apply the relevant assessment methods of coronary atherosclerosis to the study of intracranial atherosclerotic stenosis (ICAS), aiming to improve the precision diagnosis and treatment of cerebrovascular diseases (4). This cross-organ, cross-disciplinary research path further expands the comprehensive prevention and control ideas for vascular diseases and provides a solid theoretical foundation for personalized and precise medicine (Figure 1).

## 2 Vascular imaging

### 2.1 Intravascular ultrasound

Intravascular ultrasound (IVUS) technology is an intravascular catheter-based imaging technique that can comprehensively acquire relevant information about vascular lesions. It holds significant value in the diagnosis and treatment of cardiovascular and cerebrovascular diseases. As a real-time and precise imaging technology, IVUS plays a crucial role in measuring lumen diameter, understanding vessel wall structure, identifying plaque characteristics, and guiding stent placement. Park et al. utilized IVUS in a study of 112 patients with coronary artery stenosis and found that a minimum lumen area (MLA) of ≤4.5 mm^2^ had a sensitivity and specificity of 77 and 82%, respectively, for identifying patients with a fractional flow reserve (FFR) of ≤0.80 (10). This parameter can be employed as a significant indicator for identifying functionally critical moderate coronary artery stenosis. Additionally, IVUS-guided percutaneous coronary intervention (PCI) has been shown to increase post-implantation MLA, reduce stent restenosis rates, and long-term follow-up results have confirmed the effective reduction of major adverse cardiovascular events (MACE) occurrence and target vessel revascularization, significantly improving patient prognosis (11, 12).

### 2.2 Advancements in the research of cerebrovascular disease

#### 2.2.1 Vascular stenosis assessment

Atherosclerotic plaque is a significant contributor to ischemic stroke, and early identification of atherosclerosis is crucial for the prevention and treatment of cerebrovascular diseases. Plaques lead to lumen narrowing, prompting compensatory dilation in some vessels, known as vascular remodeling. Positive remodeling involves an increase in the external elastic membrane area while the lumen diameter remains unchanged or increases. Compared to DSA, IVUS can accurately identify the structure of the artery wall, thus holding greater application value in the identification of early lesions. Furthermore, research has demonstrated that IVUS can provide a more precise assessment of the extent and nature of internal carotid artery stenosis in patients with transient ischemic attack (TIA), thereby offering a crucial basis for clinical decision-making (13). Building upon the precise assessment of the degree of artery stenosis by IVUS, the discussion by Meyers et al. regarding artery stenosis caused by internal plaque bleeding has advanced the further application of IVUS in this field (14). Therefore, we believe that IVUS plays a significant role in the diagnosis and treatment of intracranial atherosclerotic stenosis and holds important application value in assessing arterial stenosis and its etiology.

#### 2.2.2 Plaque assessment

In the assessment of plaque vulnerability, virtual histology intravascular ultrasound (VH-IVUS) technology analyzes radiofrequency signals from plaques and categorizes them into four types: fibrous, fibro-fatty, calcified, and necrotic core, marked with four distinct colors: deep green, light green, white, and red, respectively (15). A study evaluating carotid atherosclerotic plaque with virtual histology confirmed that VH-IVUS demonstrated high consistency in identifying plaque components compared to histological results (16). Moreover, certain lesion characteristics assessed by IVUS, such as thin-cap fibroatheroma and necrotic tissue content, have been identified as independent predictors of adverse cerebrovascular events (16). A single-center prospective study, which observed carotid plaque features via IVUS, found that patients with a history of cerebrovascular disease had a higher proportion of necrotic tissue within the plaque compared to those with asymptomatic and symptomatic carotid artery stenosis (23.5% ± 10.7% vs. 18.9% ± 8.2 and 18.7% ± 9.5%, *p* = 0.11) (17). Furthermore, Gonzalez et al. further demonstrated the high instability of thin-cap fibroatheromas, through VH-IVUS technology. As the necrotic core of the plaque approaches the lumen or the amount of calcification increases, the risk of plaque rupture further rises (18).

#### 2.2.3 Guiding stent placement

Wehman et al. reported a study confirming the feasibility and safety of IVUS-assisted intracranial vascular interventions (19). In the first case, clinical practitioners identified a dissected false lumen in the left internal carotid artery using IVUS, ensuring complete stent coverage. In the second case, a patient underwent stent placement for high-grade basilar artery restenosis. IVUS was utilized to identify the composition and morphological features of the restenosis and assist in determining the safety of lesion stenting. Additionally, Chiocchi et al. suggested that IVUS can provide detailed information about the arterial lesion for the treatment of unstable plaques or cases requiring morphological assessment to choose an appropriate stent, especially those with a high risk of embolization (20). Furthermore, Tanaka et al. found that IVUS could detect situations where DSA was negative but there were issues such as poor stent apposition or inadequate stent expansion, providing a basis for operators to take timely remedial measures. Therefore, IVUS has proven to be a valuable tool in clinical practice, contributing significantly to our understanding of intracranial circulatory vascular diseases (21).

## 3 Optical coherence tomography

Optical coherence tomography (OCT) is a high-resolution imaging technology that utilizes the principle of optical interference. By measuring the reflection and echo delay time of light waves, it generates high-resolution cross-sectional images. OCT can be divided into Time-Domain OCT (TD-OCT) and Frequency-Domain OCT (FD-OCT). In the early stages, intravascular OCT was applied in the diagnosis and treatment of cardiovascular diseases such as acute coronary syndromes. It not only accurately assesses plaque stability but also guides and optimizes PCI, contributing to precise patient treatment (22, 23). Research comparing OCT-guided and angiography-guided PCI found no significant statistical difference in postoperative Minimum Stent Area between the two approaches (24). However, further studies suggested that OCT-guided intervention significantly reduces the stent implantation rate in patients with ST-segment elevation myocardial infarction (25). Therefore, OCT is considered to be of crucial value in optimizing interventional treatment plans and achieving precision therapy.

### 3.1 Advancements in the research of cerebrovascular disease

#### 3.1.1 Vascular stenosis assessment

OCT, with its high resolution, can accurately differentiate tissue structures. In the study by Mathews et al., OCT was used for endovascular imaging of the intracranial segment of the internal carotid artery, and the vascular structure images obtained by OCT showed a strong correlation with those obtained by tissue sections (26). This capability to visualize the layered structure of blood vessels holds potential value for the early diagnosis of cerebrovascular diseases. Feng et al. further investigated stenotic arteries in the V4 segment of the vertebral artery and concluded that OCT can measure the stenosis cross-sectional area more accurately than DSA, establishing it as a reliable tool for assessing the degree of artery stenosis (27). Additionally, Yang et al. extended the application of OCT to evaluate more complex anterior circulation ICAS before and after circulation, further confirming the feasibility and safety of OCT in the diagnosis and evaluation of cerebrovascular stenosis (28). This underscores the importance of OCT as a tool for the diagnosis and treatment of cerebrovascular diseases. In the study, 35 out of 36 cases included had lesions detected by FD-OCT imaging catheters. Ultimately, FD-OCT imaging was successful in 27 patients, where the target lesions were completely visible and no symptomatic complications were observed. These studies emphasize the significance and feasibility of OCT in the diagnosis and risk assessment of cerebrovascular diseases, highlighting its importance as a tool in the diagnosis and treatment of ICAS.

#### 3.1.2 Plaque assessment

In the assessment of plaque vulnerability, vulnerable plaques are associated with the risk of ischemic stroke. The main pathological features of vulnerable plaques include a thin fibrous cap, a large lipid-rich necrotic core, and intraplaque hemorrhage (29). The high resolution of OCT is helpful in revealing plaque characteristics, making it potentially valuable for accurate assessment of plaque rupture and risk stratification (30). Research utilizing OCT for plaque analysis has found that a fibrous cap thickness of 130 μm may be a threshold for plaque rupture in patients with symptomatic carotid artery stenosis (31). Additionally, Shibutani et al. demonstrated that quantitative analysis of OCT signal attenuation (the degree to which signal intensity decreases with increasing distance from the luminal surface) can effectively identify vulnerable plaques, with attenuation rates greater than 2.3 and 3.1% indicating LRNC and IPH, respectively (32). Another study using OCT to assess plaques by reflecting on neovascularization within plaques found that the quantity of neovessels within the plaque, specifically ≥6, is a reliable threshold for rapid plaque progression (33). Therefore, intravascular OCT is an ideal tool for plaque assessment, aiding in the evaluation of plaque vulnerability.

#### 3.1.3 Guiding stent placement

OCT can provide microscopic structural information about lesions and assess the relationship between stents and vessel walls, guiding interventional treatments. Yan et al. reported a case of vertebral artery stenosis where OCT, in addition to identifying the stenosis, reflected the thrombotic conditions of atherosclerotic plaques and the distal narrowing, providing more information for treatment planning (34). A study by Given et al. reported a case of a patient who experienced fossa ischemia after undergoing vertebral artery stenting (35). The study emphasized the efficacy of FD-OCT in visualizing the interaction between the stent and the vessel wall, providing valuable insights into the mechanism of stent thrombosis and the potential for eliminating residual flow-limiting stenosis. During stent implantation, plaque prolapse between stent struts may occur, which is associated with ipsilateral ischemic brain injury. OCT can accurately reflect plaque prolapse within the stent and guide the use of overlapping stents to reduce plaque prolapse (36). Donato et al. found that open-cell design stents are more prone to plaque prolapse, through OCT assessment of patients after different types of stent placement (37). These findings can influence the choice of the appropriate stent type during interventional treatment.

## 4 High resolution MR vessel wall imaging

High resolution MR vessel wall imaging (HR-VWI) is an MRI technique capable of non-invasively displaying luminal and wall lesions in blood vessels. It reveals the lumen and wall lesions of blood vessels, providing information on plaque distribution, morphology, and vulnerability for the assessment of plaque risk. In the context of coronary artery lesions, the T1-shortening effect resulting from features such as intra-plaque hemorrhage, thrombus, and lipid core is utilized to assess the stability of coronary artery plaques (38). The presence of high-intensity signal plaques on non-contrast-enhanced T1-weighted inversion-recovery (T1W-IR) sequence images may reflect the vulnerability of plaques and may be associated with intra-coronary thrombus (39, 40). A correlation exists between high-intensity signal plaques and intra-plaque hemorrhage and lipid-rich plaque (41, 42).

### 4.1 Advancements in the research of cerebrovascular disease

HR-VWI can detect signs of plaque thickening, abnormal enhancement, wall structure remodeling, and intra-plaque hemorrhage, which are considered indicators of high-risk plaques. Intra-plaque hemorrhage is associated with symptoms and may serve as a predictive factor for ischemic stroke (43). Some studies have utilized HR-VWI to detect intra-plaque hemorrhage in intracranial artery plaques, finding that the T1 signal intensity of intra-plaque hemorrhage is 150% higher than that of surrounding tissue, and symptomatic patients have a higher detection rate of intra-plaque hemorrhage (42, 44). Research has demonstrated that HR-VWI has clinical value in identifying intracranial artery atherosclerosis intra-plaque hemorrhage and may predict the risk of stroke. Furthermore, some studies propose that the enhanced information provided by HR-VWI may indicate plaque vulnerability. Skarpathiotakis et al. investigated the presence of enhanced intracranial artery atherosclerotic plaques in vessels supplying the infarcted area to analyze the connection between plaque enhancement and cerebrovascular events, through HR-VWI analysis (45). The results showed a strong pathological enhancement of intracranial artery atherosclerotic plaques in all vessels supplying the stroke area within 4 weeks of a stroke. The study suggests that HR-VWI can identify enhanced plaques in intracranial artery atherosclerosis, assess plaque risk, and predict the incidence of cerebrovascular events, thereby guiding clinical decisions. However, some studies indicate that plaque enhancement may not be the sole independent influencing factor, and the degree of stenosis in the target vessel may impact the relationship between plaque enhancement and cerebrovascular events (46). Therefore, although HR-VWI holds certain value in assessing plaque risk, extensive research is still needed to determine the inherent connection between HR-VWI plaque characteristics and cerebrovascular events.

## 5 Hemodynamics in ICAS

### 5.1 Fractional flow reserve

Fractional flow reserve (FFR) is defined as the ratio of the maximum blood flow in a coronary artery lesion to the maximum blood flow in the same vessel under healthy conditions (47). Since myocardial blood flow is proportional to perfusion pressure, FFR can be crudely described as the ratio of the distal pressure (Pd) in the narrowed vessel to the pressure at the arterial ostium (Pa) at maximum hyperemia, FFR = Pd/Pa (48). In 1993, Pijls et al. first proposed the use of FFR to assess coronary flow reserve for guiding treatment decisions (47). The results indicated that patients with FFR < 0.75 had evidence of reversible myocardial ischemia in at least one non-invasive test, suggesting the need for aggressive treatment (49). Conversely, patients with FFR > 0.75 were considered to have good hemodynamic status, and PCI was deferred, with no significant impact on long-term prognosis (50, 51). Subsequent FAME trials and follow-up studies have demonstrated the benefits of FFR-guided PCI, both in the short and long term (52, 53).

### 5.2 Advancements in the research of cerebrovascular disease

Due to the absence of the pulsatile status of the heart and the inability to use adenosine in cerebral arteries, the pressure ratio measured across lesions, analogous to coronary FFR, is referred to as cerebral blood flow fraction (Fractional Flow, FF) in this context.

### 5.3 FF measured using a pressure wire

The measurement of cerebral FF using a pressure wire is a direct method that involves introducing a pressure wire into the stenotic vessel, measuring pressures before and after the lesion, and calculating the FF value. Some researchers argue that FF serves a similar function as FFR in blood evaluation and have confirmed its safety and feasibility in cerebral vasculature. Severe ICAS cases have been found to exhibit a widespread decrease in FF, with varying degrees of improvement observed after interventional treatment (54, 55). Although there is currently no large-scale clinical trial to establish the critical FF value for guiding ICAS intervention, small-sample clinical studies suggest that an FF value of 0.7 may be a critical threshold. Based on the application of FF in the coronary artery field, Han et al. explored the feasibility of using pressure gradient measurements to assess the severity of intracranial large artery stenosis. The study included 12 cases of intracranial large artery stenosis (including intracranial internal carotid artery, M1 segment of the middle cerebral artery, intracranial vertebral artery, and basilar artery). These patients had stenosis rates exceeding 40% and a history of transient ischemic attack (TIA) or non-disabling stroke. The study measured the pressure gradients proximal and distal to the stenotic site before and after percutaneous transluminal angioplasty and stenting (PTAS), using Pd/Pa ≤ 0.7 as the critical threshold for guiding intervention. Results from a 6-month follow-up showed that, except for one patient with FF <0.7 who experienced TIA without intervention, the remaining patients had no stroke events (55). Another study included 18 patients with intracranial atherosclerotic stenosis, encompassing 19 stenotic vessels. The study used a pressure wire to measure the pressure and calculate the pressure ratio before and after endovascular intervention, comparing it with diameter stenosis and Tmax (an indicator of cerebral ischemia) (56). The study suggested that the pressure ratio might be related to perfusion status, and when using Tmax>6 s as the critical threshold, an FF value of 0.675 might be the optimal guiding value (56). The increasing interest among neurointerventionalists and the intention to conduct large-scale multicenter clinical studies underscore the evolving significance of FF in guiding ICAS intervention (57, 58).

### 5.4 Non-invasive FF detection based on imaging

Non-invasive imaging techniques for measuring FF can reduce the risks associated with the use of pressure wires. A study demonstrated that hemodynamic analysis based on CTA images can provide FF values similar to those obtained from DSA and can reflect the status of patients’ leptomeningeal collateral circulation (59). In a cohort of 245 ICAS patients receiving medical treatment, FF values and wall shear stress (WSS) based on CTA were measured, and a one-year follow-up revealed an association between low FF, high WSS, and recurrent stroke within 1 year. Further research indicated that the combination of low FF and high WSS posed a risk factor for poor prognosis compared to normal FF and WSS. Some researchers attempted to calculate FF using the signal intensity ratio (SIR) inside the lumen before and after stenosis based on time-of-flight magnetic resonance angiography (TOF-MRA). Their study found that FF ≤ 0.9 was closely related to reduced perfusion after stenosis (60). Another study found a strong correlation between decreased signal intensity in the distal segment of stenosis and core infarction and recurrent strokes (61). Non-invasive imaging techniques for measuring FF have proven to be feasible, with the major advantage of being non-invasive. However, their accuracy and clinical value require further research and confirmation, given the high demands on image quality and post-processing.

### 5.5 FF stimulated by hemodynamic analysis

Blood flow dynamics analysis based on computer simulations can also calculate FF values without the need for pressure wires by simulating the reconstructed vessel geometry and hemodynamic changes. Huang et al. utilized hemodynamic analysis software (AngioPlus Core, Pulse Medical Imaging Technology, Shanghai, China) to perform quantitative flow ratio (QFR) assessment based on single-image analysis (62). This method evaluates hemodynamic alterations caused by luminal stenosis, and the results showed a high consistency between QFR and FFR. Another study employed AccuFFicas software (AccuFFicas V1.0; ArteryFlow Technology, Hangzhou, China) to calculate FF and compared it with FF measured using pressure wires. The findings suggested a high level of consistency between the two methods, with an optimal area under the curve (AUC) of 0.986 when FF was ≤0.75 (63). These research outcomes indicate the feasibility of wire-free FF measurements.

Wu et al. utilized FF to assess the functional status of patients with ICAS (64). The study included 56 symptomatic ICAS patients who underwent endovascular treatment. FF was measured using a pressure wire and analyzed in conjunction with watershed infarct areas derived from DWI analysis and tissue perfusion status assessed by CTP. The results indicated that FF has significant predictive value for evaluating the functional status and prognosis of ICAS patients.

### 5.6 Wall shear stress

Prolonged exposure to high wall shear stress (WSS) can lead to abnormal endothelial cell function, triggering inflammatory responses and promoting the formation of atherosclerotic plaques. Under the combined mechanical effects of high WSS and plaque rupture (PR), vulnerable plaques are more likely to occur. This condition increases the risk of plaque rupture and intraplaque hemorrhage, contributing to the development of in-situ thrombus formation or embolic events (65, 66).

In the study, which included 245 patients with ICAS, hemodynamic analysis based on CTA data revealed an association between high WSS and the risk of recurrent stroke in patients with moderate to severe stenosis. When combined with PR detection, it was found that patients with high WSS and low PR had a higher risk of stroke recurrence (66). However, a study by Lan et al.65 found that, under aggressive medical treatment, most symptomatic ICAS patients experienced lesion regression or maintained stable luminal narrowing within 1 year after a stroke. Moreover, locally elevated WSS and a larger area of high WSS were independently associated with the regression of luminal narrowing within 1 year (67). Therefore, the role of WSS in ICAS requires further investigation. Additionally, Liu et al. used a computational fluid dynamics model based on CTA to simulate the effects of different stent geometries in ICAS after stent placement. They observed that various stent shapes could lead to differences in local hemodynamics Stents with partial apposition or eccentric narrowing were associated with increased local WSS (68). Real-time hemodynamic analysis can be employed to evaluate the patency of ICAS stents, providing guidance for interventional treatments.

Additionally, Liu et al. conducted a systematic analysis of the clinical significance of hemodynamics in ICAS, further substantiating the clinical value of hemodynamic parameters (69). The study analyzed the pressure and WSS at the proximal and distal ends of lesions in ICAS patients with stenosis ranging from 50 to 99%. They concluded that FF and the stenotic-throat to prestenotic WSS ratio (WSSR) are predictive indicators for assessing the severity and prognosis of ICAS patients. Both lower FF and higher WSSR are associated with the severity of cerebrovascular disease, providing reference metrics for evaluating the impact of endovascular treatment on focal hemodynamics.

## 6 Novel devices in ICAS

In the coronary field, the application of technologies such as IVUS and OCT allows clinical practitioners to visualize the situation of stents in blood vessels, achieving a visual assessment of the treatment effects of different stents. This has prompted the development of stents with characteristics such as complete plaque coverage, high vascular compliance, and good adherence to the vessel wall. Currently, stents can be classified into bare-metal stents (BMS), drug-eluting stents (DES), and bioresorbable stents. However, the use of metal as a foreign object in the body leads to long-term stimulation at the affected site, causing local vascular imbalance, vascular wall inflammation, intimal hyperplasia, and a significant disappearance of vasomotor responses in the stented segment, ultimately leading to vascular restenosis and thrombosis. Even the latest DES cannot permanently address the limitations of metal stents, such as target lesion revascularization and stent thrombosis (70). To overcome these limitations, bioresorbable stents have been designed as vascular support technology, allowing structural and functional restoration of the vessels after initial stent absorption (71). A study involving 20 patients with bioresorbable stents and 18 with drug-eluting stents found that the vascular reactivity in patients with bioresorbable stents was higher than that in patients with DES after 3 years (77.8% vs. 25.0%, *p* = 0.008, and 61.1% vs. 18.8%, *p* = 0.018) (72). The latest prospective multicenter human study on the use of the third-generation coronary sirolimus-eluting magnesium scaffolds in the treatment of coronary stenosis suggests good safety and efficacy at the 1-year follow-up, with no scaffold-related adverse events reported (73). Therefore, bioresorbable stents may be a solution to the limitations of BMS and DES.

### 6.1 Advancements in intracranial stents for ICAS

Following the development trajectory of coronary stents, intracranial stents for ICAS are also continuously evolving. From the initial development and marketing of the Wingspan intracranial self-expanding stent in 2005, which participated in the SAMMPRIS clinical trial with less-than-ideal clinical results and a significantly higher rate of endpoint events than medical therapy (1, 74), to the CASSISS study suggesting that the effect of Wingspan stent placement is not inferior to medical treatment (3). Since 2019, the development of Neuroform EZ, Enterprise EP BMS and other microcatheter-released BMS, with the ability to be released through extremely thin microcatheters, has stronger capabilities to navigate through complexly curved vessels compared to the original bare-metal ICAS-specific stents, providing superior safety during the perioperative period (75, 76). However, BMS still face the issue of in-stent restenosis (ISR), with reported restenosis rates as high as 15–30% within 1 year post stent placement (75, 76).

To reduce this incidence, DES have been introduced, with a recent prospective study comparing the NOVA DES with the Apollo BMS in ICAS patients showing NOVA DES significantly reduced the incidence of 1-year ISR (9.5%) and markedly lowered the rate of 1-year ischemic stroke recurrence and death (8.4%) (77). On this basis, a prospective multicenter single-group target value study evaluating the effectiveness and safety of a self-expanding intracranial DES has been completed for enrollment and is currently in the clinical follow-up phase. This self-expanding DES offers better radial support, better conformability, and enhanced operational safety, potentially further reducing the incidence of complications (NCT05217459). Intracranial bioresorbable stents are currently in the exploration phase, and it is believed that with the continuous deepening of the concept of “panvascular medicine “, novel stents applied to coronary lesions, after improvement, will be adapted for ICAS.

## 7 Conclusion and outlook

In summary, based on the concept of “panvascular medicine “, many examination methods and new devices have been applied to ICAS, providing a more comprehensive perspective and integrated assessment in the evaluation of ICAS. This helps identify systemic risk factors, assess multi-site vascular lesions, comprehensively evaluate microcirculation function, and formulate personalized treatment strategies. However, it is essential to note that some shortcomings of these examination methods limit their widespread application in cerebrovascular diseases. For instance, the index of microcirculatory resistance (IMR), which can directly reflect the reserve function of the capillary bed supplying the myocardium by measuring temperature, may accurately assess the degree of myocardial damage after coronary lesions. Still, the current temperature probes cannot reflect changes in temperature in cerebrovascular diseases due to the more tortuous nature of cerebral vessels. Although OCT and IVUS have high resolutions, their poor penetration and limitations in instrument diameter may pose challenges for comprehensive imaging of some complex cerebrovascular lesions. Additionally, researchers are exploring the use of biodegradable polymer materials such as polylactic acid (PLA) and polycaprolactone (PCL) to enhance the biocompatibility of bioresorbable stents for intracranial vessels while optimizing their supportive capabilities. However, given the complexity of intracranial vasculature, the safety and efficacy of bioresorbable stents still require further clinical investigation. With advancements in 3D printing technology, the customization of bioresorbable stents to fit individual patients’ vascular morphology has become possible, potentially improving treatment precision and outcomes. This progress holds promise for further enhancing patient prognosis and reducing the incidence of in-stent restenosis. Nevertheless, with further technological development, the value of the panvascular disease concept in the diagnosis and treatment of ICAS will continue to be explored and widely applied. A multidimensional preoperative assessment and precise diagnosis and treatment strategy are the direction of ICAS development.
